# Azole Resistance of *Aspergillus fumigatus* in Immunocompromised Patients with Invasive Aspergillosis

**DOI:** 10.3201/eid2201.151308

**Published:** 2016-01

**Authors:** Jan W.M. van der Linden, Maiken C. Arendrup, Willem J.G. Melchers, Paul E. Verweij

**Affiliations:** Radboud University Medical Center, Nijmegen, the Netherlands (J.W.M. van der Linden, W.J.G. Melchers, P.E. Verweij);; Statens Serum Institut, Copenhagen, Denmark (M.C. Arendrup)

**Keywords:** *Aspergillus*, drug resistance, fungal, fungi, azoles, the Netherlands

**To the Editor:** Alanio et al. comment that the prevalence of azole-resistant *Aspergillus* disease may differ, depending on location of the hospital where patients are admitted and the patients’ underlying disease (*1*). Determining local or regional epidemiology, especially in areas where azole-resistant isolates are found in the environment, is indeed important. These isolates commonly harbor the TR_34_/L98H or TR_46_/Y121F/T289A resistance mechanism. Patients may inhale azole-resistant spores in the air and subsequently develop azole-resistant disease, even when they have never been treated with azoles (*2*). Although risk for inhalation of azole-resistant *Aspergillus* spores arguably might be similar for all patients, surveillance of *Aspergillus* isolates in the Netherlands indicates that resistance rates vary among hospitals. When all *A. fumigatus* isolates cultured from patients were investigated for azole resistance, resistance rates in the Netherlands ranged from 4.3% to 19.2% in 2013 and 3.8% to 13.3% in 2014 (*3*). The highest and lowest resistance rates were found in hospitals only 39 km from each other, supporting the observation made by Alanio et al. about variations in prevalence of azole-resistant *Aspergillus* disease (*1*).

More detailed surveillance is required to determine if local treatment guidelines should be reassessed. Two recent studies in the Netherlands investigated the risk of azole-resistant invasive aspergillosis in high-risk populations. One study conducted in a 33-bed tertiary-care university hospital intensive-care unit (ICU) showed that 26% of culture-positive patients with presumed invasive aspergillosis harbored azole-resistant isolates, a proportion 14% higher than that found in other departments in the hospital (p = 0.06) (*4*). The second study, which investigated azole resistance in the primary routine culture (including respiratory cultures) of 105 ICU and hematology patients, showed that the resistance rate (24.6%) for hematology patients was higher than the rate (4.5%) for ICU patients (*5*). Other countries have also reported higher prevalence of resistance in high-risk populations than in other populations. 

One problem with assessing prevalence of azole resistance is that the recovery of *A. fumigatus* in culture may vary considerably among different patient groups. A recent audit in our hematology department over the past 5 years indicated that *A. fumigatus* was cultured in only 35% of patients who underwent bronchoalveolar lavage as part of a diagnostic work-up for pulmonary infection (P.E. Verweij, unpub. data). This outcome indicates that in culture-negative patients, presence of azole resistance will be missed.

In agreement with Alanio et al. (*1*), recent studies show a need to determine frequency of azole resistance at the hospital level and within different patient groups or departments. Although surveillance of unselected clinical cultures provides resistance rates at a national level and offers information about the epidemiology of resistance mechanisms, regular audits in specific patient populations are warranted to determine the frequency of azole resistance among different risk groups. These audits will enable clinicians to determine whether reassessment of azole monotherapy as a primary treatment option is necessary. Given the low and variable rates of positive cultures, culture-negative patients should also be included in azole-resistance surveillance programs. 
